# Differential Immune Responses to Hemorrhagic Fever-Causing Arenaviruses

**DOI:** 10.3390/vaccines7040138

**Published:** 2019-10-02

**Authors:** Emily Mantlo, Slobodan Paessler, Cheng Huang

**Affiliations:** 1 Department of Pathology, University of Texas Medical Branch at Galveston, Galveston, TX 77555, USA; ekmantlo@utmb.edu (E.M.); slpaessl@utmb.edu (S.P.); 2 Department of Microbiology and Immunology, University of Texas Medical Branch at Galveston, Galveston, TX 77555, USA

**Keywords:** arenavirus, hemorrhagic fever, immunity, interferon, innate sensing, Lassa virus, Junín virus, Machupo virus

## Abstract

The family *Arenaviridae* contains several pathogens of major clinical importance. The Old World (OW) arenavirus Lassa virus is endemic in West Africa and is estimated to cause up to 300,000 infections each year. The New World (NW) arenaviruses Junín and Machupo periodically cause hemorrhagic fever outbreaks in South America. While these arenaviruses are highly pathogenic in humans, recent evidence indicates that pathogenic OW and NW arenaviruses interact with the host immune system differently, which may have differential impacts on viral pathogenesis. Severe Lassa fever cases are characterized by profound immunosuppression. In contrast, pathogenic NW arenavirus infections are accompanied by elevated levels of Type I interferon and pro-inflammatory cytokines. This review aims to summarize recent findings about interactions of these pathogenic arenaviruses with the innate immune machinery and the subsequent effects on adaptive immunity, which may inform the development of vaccines and therapeutics against arenavirus infections.

## 1. Introduction

Arenaviruses are enveloped, negative-sense, single-stranded RNA (ssRNA) viruses [1]. The family *Arenaviridae* currently consists of four genera, *Mammarenavirus*, *Reptarenavirus*, *Hartmanivirus*, and *Antennavirus* [2,3]. With the exception of the trisegmented *Antennavirus* genus, arenavirus genomes are bi-segmented, with one large (L) segment of around 7.2 kb and one small (S) segment of around 3.4 kb. Each segment contains two open reading frames (ORFs) encoding two gene products in opposite orientation, allowing the virus to assume an ambisense coding strategy. The two ORFs are separated by a highly structured intergenic region (IGR) that functions to terminate viral RNA transcription [4]. The conserved termini regions of each genomic segment form pan-handle structures and mediate viral RNA replication and transcription [5,6]. The S segment encodes the viral glycoprotein (GP) precursor, which is post-translationally cleaved into stable signal peptide (SSP) and mature GP1 and GP2 [7,8,9]. All three of these cleaved products form the glycoprotein complex and are incorporated into virions, with GP1 and GP2 forming the spikes on the surface of virions that bind to host receptors and mediate cell entry [10]. The S segment also encodes the nucleoprotein (NP), which is the most abundant viral protein produced during infection and the major structural component of the nucleocapsid [1]. The L segment encodes the RNA-dependent RNA polymerase L protein and a small, zinc finger protein (Z), which acts as the arenavirus matrix protein that drives the assembly and budding of virus particles [11,12,13].

Within the family *Arenaviridae*, all human pathogens are members of the *Mammarenavirus* genus [2]. Mammarenaviruses are further separated into two groups based on geography and phylogeny: the Old World (OW) arenaviruses and the New World (NW) arenaviruses [14]. Lassa virus (LASV) is endemic in West Africa and is therefore classified as an OW arenavirus. The prototypic lymphocytic choriomeningitis virus (LCMV) is also classified as an OW arenavirus based on similar phylogeny [15]. Meanwhile, NW arenaviruses are endemic to South America and can be further divided into four clades (A–D). Clade B contains all the pathogenic NW arenaviruses, including Junín (JUNV) and Machupo viruses (MACV), the causative agents of Argentine hemorrhagic fever (AHF) and Bolivian hemorrhagic fever (BHF), respectively. Notably, clade A contains the prototypic Pichinde virus that, while non-pathogenic to humans, causes hemorrhagic disease in rodents that is similar to Lassa fever (LF) in humans [16,17].

*Mammarenavirus* (with the exception of Tacaribe virus) are rodent-borne viruses, which usually infect specific rodent species. Therefore, the geographic distribution of each arenavirus is defined by the range of the habitat of its host rodent species. *Mastomys natalensis*, the reservoir for LASV, is found across much of Africa, though most LASV infections occur in *M. natalensis* monophylogenetic group A–I in West Africa [18,19]. NW arenaviruses likewise each primarily infect a single species of rodent in the Americas. Arenaviruses often persistently infect their natural hosts without overt disease signs and are shed via excreta from infected animals. The transmission of pathogenic arenaviruses to humans occurs largely through aerosol exposure to rodent excreta or consumption of rodent meat [1,20]. Most infections occur in a rural setting, often during cyclical outbreaks. However, nosocomial transmission of LASV, JUNV, and MACV has been reported [1,21,22].

Within endemic areas, both OW and NW arenaviruses are responsible for significant human disease. Among the highly pathogenic arenaviruses, LASV is the most prevalent and clinically important, with an estimated 100,000–300,000 infections and 5000 deaths in West Africa each year [23]. While most LASV infections are asymptomatic, severe LF can have case fatality rates ranging from 9.3–18% among hospitalized patients [24]. For pathogenic NW arenaviruses (JUNV and MACV), the case fatality rates can be as high as 15–35% [25,26].

In addition to the severe acute disease and high mortality rates in humans, long-term sequelae are common but often neglected among survivors. Patients recovering from AHF and BHF often experience a protracted convalescence period, with hair loss and neurological symptoms such as dizziness and headaches lasting up to several months after the acute infection [1,25,26]. Neurological sequelae have also been reported in LF cases [27]. Recently, the prevalence and impact of LASV-induced hearing loss is becoming increasingly recognized as a significant social and economic burden in affected areas [28]. Approximately 33% of LF survivors develop unilateral or bilateral sudden-onset sensorineural deafness that may be permanent [29]. The exact mechanisms behind the development of long-term sequelae after infection by highly pathogenic arenaviruses remain to be determined, but cell-mediated immunity may be involved. Currently, vaccines and treatments are very limited for these hemorrhagic fever-causing arenaviruses. The World Health Organization has listed LF in the Blueprint list of priority diseases for which there is an urgent need for accelerated research and development.

## 2. Immune Response to Hemorrhagic Fever-Causing Arenaviruses

**Figure 1:**
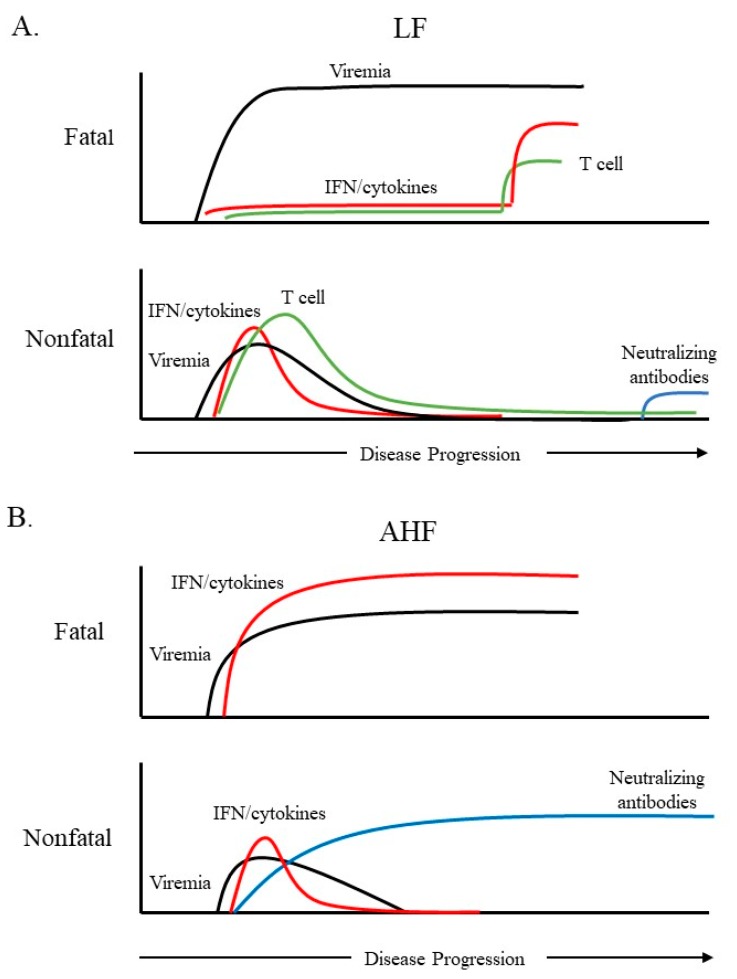
A schematic diagram of the immune response over the course of fatal or nonfatal cases of Lassa fever (LF) or Argentine hemorrhagic fever (AHF). (**A**). Fatal cases of LF are characterized by a lack of interferon (IFN) and pro-inflammatory cytokines throughout most of the infection. A cytokine spike late in infection has been reported in some fatal LF cases. Survival and Lassa virus (LASV) clearance are correlated with a robust T-cell response as well as higher IFN levels early in infection. Neutralizing antibodies are not detectable until late in convalescence. (**B**). Extremely high levels of serum IFN/pro-inflammatory cytokines are observed in fatal cases of AHF. Survival is correlated with lower levels of IFN/cytokines, which decrease as viremia subsides. AHF survivors often develop high neutralizing antibody titers during the acute phase of illness.

While highly pathogenic OW and NW arenaviruses cause severe diseases in humans, the innate immune responses are remarkably different (Figure 1). Despite high unchecked levels of viremia, severe LF cases are generally characterized by profound immunosuppression, including a weak or delayed Type I interferon (IFN-I) and pro-inflammatory cytokine response particularly in the early stage of illness [30,31]. In contrast, pathogenic NW arenavirus infection stimulates a robust IFN-I response in patients. In AHF cases, serum IFN-α levels have been reported to peak at 64,000 IU/mL, one of the highest circulating interferon levels recorded for any viral disease [32]. Elevated levels of IL-6, IL-8, IL-10, and TNF-α are also observed in the serum of AHF patients. High IFN-I and cytokine levels are correlated with the severity and outcome of AHF disease [32,33]. Also, there is evidence that the IFN response may contribute to thrombocytopenia in AHF patients [34].

Pathogenic OW and NW arenavirus infections also induce differential adaptive immune responses, which could be linked to the profound difference in the innate response (Figure 1). The host innate immune response not only acts directly to counteract virus infection, but also motivates and regulates virus-specific, adaptive immune responses that provide long-term protection. Dendritic cells (DC) and macrophages, which present viral antigens to T cells, are essential components of both the innate immune response and the adaptive immune response. These cells are also the early targets in arenavirus infection. LASV efficiently infects monocyte-derived dendritic cells (moDC) and macrophages without stimulating IFN and cytokine responses. Consequently, LASV-infected moDCs and macrophages fail to activate T cells, which explains the delayed cellular immune response [30,35,36]. The nonpathogenic OW Mopeia virus (MOPV) is genetically closely related to LASV and has been used as a control to study the pathogenesis of LASV. In contrast to LASV, MOPV infection induces strong IFN and cytokine responses in moDCs and macrophages [37], leading to T cell activation. In the host, myeloid dendritic cells (mDCs) are specialized in antigen presentation and the induction of T cell responses. A recent study has identified that LASV and MOPV infection of mDCs ex vivo are abortive [36] due to IFN and cytokine responses. Blocking IFN enables productive infection for both LASV and MOPV in mDCs. Interestingly, in a mDC and autologous T cell coculture model, mDC activation and the IFN response are remarkably reduced and delayed in LASV infection, while mDC activation and the IFN-I response in MOPV infection are increased and prolonged [36]. Consistently, LASV-infected mDCs do not activate T cells, while MOPV-infected mDCs efficiently activate T cells. The mechanism underlying the apparent negative regulation of mDC activation and IFN response by T cells in LASV infection remains unknown. It is also unclear if other cell types, e.g., NK cells, might be present in the coculture and contribute to the phenotype. Plasmacytoid dendritic cells (pDCs) are an important IFN producer in vivo. LASV and MOPV infection of pDCs are both abortive, though LASV infection produces a less sustained and lower level of IFN-I response than MOPV [38]. Nevertheless, it has been found that LF survivors develop virus-specific CD4+ and CD8+ T cell responses during the acute stage of infection [39,40], which is associated with LASV clearance and recovery. It is generally believed that the humoral response is not important in the clearance of LASV as even LF survivors do not develop neutralizing antibodies until months after the acute infection has resolved [41]. In contrast, AHF patients develop neutralizing antibodies to JUNV during the acute phase of disease [42], which is essential for virus clearance. Furthermore, both AHF and BHF can be successfully treated using convalescent immune sera from survivors [25,43].

Research using animal models for pathogenic arenavirus infections has recapitulated many of the clinical observations. NHPs infected with LASV typically develop lymphopenia, though survival is correlated with increased numbers of CD4+ and CD8+ T cells [44,45]. Survival of NHPs from LASV infection has also been correlated with an early IFN-I response, while fatal NHP infections are characterized by a lack of type I IFN production [44,45]. A LF chimeric mouse model, in which wild-type bone marrow cells are transplanted into irradiated type I interferon receptor knockout mice, has been developed recently and reproduces the main symptoms of LF [46]. In this model, T cell-mediated immunopathology apparently plays an essential role in LF disease, highlighting the dual activity of the T-cell response during LF. More studies are required to determine whether an early T-cell response aids in LASV clearance, or whether a late and dysregulated T-cell response may actually contribute to the severity of LF. On the other hand, JUNV infection elicits a robust IFN-I response in both NHPs and guinea pigs [47,48]. As observed in LF patients, neutralizing antibodies against LASV do not form in either surviving NHPs or guinea pigs during the acute phase of infection [45,49]. In contrast, neutralizing antibodies that develop early in infection are critical for clearance of JUNV infection of guinea pigs [50]. MACV infection also induces a potent IFN response in NHPs [51] and guinea pigs [52]. In addition, as demonstrated during human cases of AHF, immune plasma is an effective treatment for AHF in NHPs [53]. These data demonstrate that hemorrhagic fever-causing OW and NW arenaviruses trigger a vastly different immune response, which has implications in viral pathogenesis as well as the development of vaccines and therapeutics.

## 3. Arenavirus Interactions with Pattern Recognition Receptors

**Figure 2:**
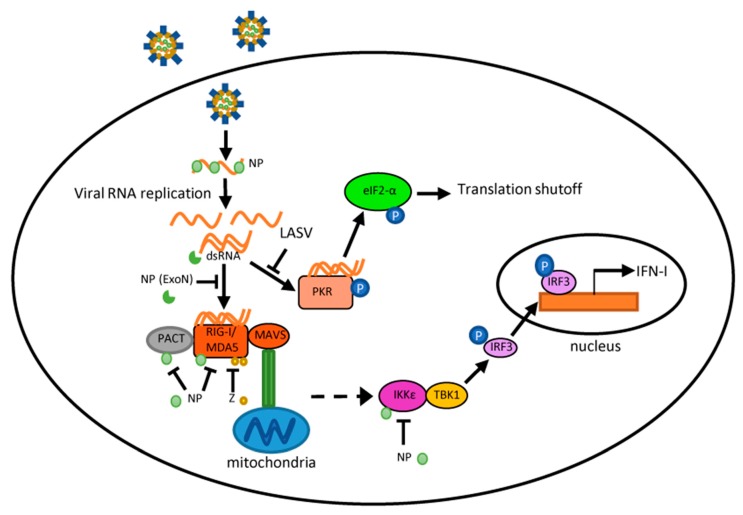
Schematic diagram of interactions between arenavirus proteins and host innate immune machinery. During arenavirus infection, viral-derived double-stranded RNA (dsRNA) molecules may be produced during viral RNA synthesis and recognized by host dsRNA sensor RIG-I-like receptors (RLRs) and protein kinase R (PKR). This may trigger a signaling cascade that leads to a Type I interferon (IFN-I) response and PKR response, respectively. The nucleoprotein (NP) exonuclease (ExoN) efficaciously degrades dsRNA in biochemistry assays. Plasmid-expressed NP and the Z of pathogenic arenaviruses also block the IFN pathway at several different points. During infection, the interaction of highly pathogenic arenaviruses with the RLR and PKR pathways differs remarkably, which is discussed in the text.

Viral RNAs often contain unique features such as 5′-triphosphate groups and double-stranded RNA structures that are distinguishable from host RNAs. In addition, viral-derived double-stranded RNA (dsRNA) molecules might form as byproducts of viral RNA replication (Figure 2). Host pattern recognition receptors (PRRs) can efficiently recognize these pathogen-associated molecular patterns (PAMPs) as danger signals of virus infection and initiate downstream signaling cascades that ultimately result in an antiviral response. Among these PRRs, the Toll-like receptors TLR7 and TLR8 are known to recognize ssRNA in endosomes, but their role during pathogenic arenavirus infection has not been determined. The majority of research into PRR recognition of arenaviruses has focused on PRRs that primarily recognize dsRNA, such as retinoic acid-inducible gene I (RIG-I) and protein kinase R (PKR) (Figure 2).

Arenavirus genomes contain non-self RNA patterns that might act as PAMPs; both ends of the genome form 5′ ppp-containing panhandle structures, while the intergenic regions naturally form hairpin loops to facilitate transcription termination [1]. In line with the JUNV-induced IFN response, a recent imaging study has revealed dsRNA accumulation during JUNV infection [54]. The dsRNA signals co-localize with the PRRs RIG-I and PKR as well as melanoma differentiation-associated protein 5 (MDA-5), strongly suggesting that these dsRNAs are recognized by and activate PRRs during arenavirus infection [54].

RIG-I has been demonstrated to mediate the IFN response to pathogenic NW arenavirus infection, while pathogenic OW LASV has seemingly evolved the ability to evade the RIG-I response. RIG-I primarily recognizes 5′ triphosphate ssRNA or dsRNA [55], then undergoes a conformational shift and ubiquitination, and further interacts and activates mitochondrial anti-viral signaling protein (MAVS) [56,57] (Figure 2). MAVS activation leads to downstream signaling cascades that induce the activation and nuclear translocation of interferon regulatory factors 3 (IRF3) and 7 (IRF7) as well as nuclear factor kappa B (NF-κB), which ultimately upregulate gene expression of IFN and pro-inflammatory cytokines. While studies have shown that transfected LASV RNA can activate the IFN-β promoter in a RIG-I-dependent manner [58], IFN-β expression is not upregulated in the context of LASV infection [59], indicating that LASV efficiently evades or inhibits the IFN response in the context of virus infection. In contrast, IFN and interferon-stimulated genes (ISGs) are upregulated in both JUNV and MACV infection [59,60]. Knockdown of RIG-I using siRNA substantially reduced IFN-β and ISG production in JUNV-infected cells, indicating that the IFN pathway is activated in a RIG-I dependent manner during JUNV infection. A recent screen of genes upregulated during NW arenavirus infection identified elevated IFN and ISG gene expression (including RIG-I) during both non-pathogenic NW Tacaribe virus (TCRV) and the Candid#1 vaccine strain of JUNV [61] infections. JUNV NP is found co-localized with RIG-I in infected cells in imaging studies [54,62], though the influence of NP on RIG-I function is still unknown. The Candid#1 strain of JUNV has also been shown to induce nuclear translocation of IRF3, indicating that the RIG-I-mediated IFN response is functional during JUNV infection [60].

Another cytosolic RIG-I-like receptor (RLR), MDA-5, has also been shown to play a role during arenavirus infection. MDA-5 binds preferentially to long dsRNA ligands and activates the IFN pathway similarly to RIG-I [55]. In JUNV infection, MDA-5 has been visualized co-localizing with dsRNA along with NP, suggesting that MDA-5 is recognizing dsRNA during JUNV infection [54]. Unlike RIG-I, MDA-5 is not required for stimulation of IFN-β upon transfection of LASV RNA [58]. A recent study with myeloid dendritic cells revealed that the non-pathogenic MOPV, but not LASV, induces upregulated MDA-5 gene expression [36]. Since MDA-5 is an ISG, the upregulation of MDA-5 is very likely mediated by the IFN response. Upregulation of MDA-5 is also observed during non-pathogenic TCRV infection as well as during Candid#1 infection [61].

Apart from the RLRs, the dsRNA-activated protein kinase R (PKR) also becomes activated following JUNV and MACV, but not LASV infection (Figure 2). PKR is ubiquitously expressed in cells and can be further transcriptionally induced by IFN [63,64]. Upon dsRNA recognition, PKR undergoes autophosphorylation and becomes enzymatically activated. PKR then phosphorylates the eukaryotic translation initiation factor 2 (eIF2α), leading to translational shutoff (Figure 2). LASV infection does not activate PKR [65], nor does it prevent PKR activation in the presence of the dsRNA analog poly (I:C), implying that LASV evades PKR detection rather than directly interfering with its kinase activity [65]. In contrast, PKR is activated during JUNV (pathogenic Romero strain) and MACV infections, concomitant with eIF2α phosphorylation and translation inhibition [65]. Expression of IFN and ISGs (e.g., OAS1 and ISG15) is augmented in PKR-deficient cells as compared with that in wild-type cells during JUNV and MACV infections. The pathogenic Romero strain of JUNV and MACV replicate slightly more efficiently in wild-type cells than in PKR-deficient cells. These results suggest that pathogenic NW arenaviruses exploit PKR activation to facilitate virus infection by attenuating ISG expression. The vaccine Candid#1 strain of JUNV also activates PKR. Interestingly, Candid#1 JUNV infection neither results in eIF2α phosphorylation nor translation inhibition [61,66], unlike the pathogenic Romero strain JUNV. Knockout of PKR does not affect Candid#1 JUNV replication. Candid#1 infection blocks poly (I:C)-induced eIF2α phosphorylation. It is worth noting that NP interacts with PKR in Candid#1 infected cells [54,66]. Whether and how JUNV NP affects the PKR pathway needs to be addressed in future studies. The non-pathogenic TCRV also induces PKR activation and phosphorylation of eIF2α [61]. Knocking out PKR facilitates TCRV infection, indicating that TCRV is susceptible to the PKR-mediated antiviral response.

## 4. Molecular Mechanisms for Arenavirus Antagonism of the IFN Response

Arenaviral NP has been identified as an IFN antagonist targeting different steps in the IFN pathway (Figure 2). Examination of the crystal structure of LASV NP has revealed a DEDDh exonuclease (ExoN) motif that preferentially targets dsRNA [67,68,69]. Purified NPs of LASV, TCRV, MOPV, and PICV have been shown to degrade dsRNA in biochemistry assays [69,70,71]. The ExoN motif is highly conserved among all arenaviruses [67,68] and has been proposed to degrade viral dsRNA produced during viral replication, preventing recognition by non-self RNA sensors such as RIG-I and MDA-5 that otherwise will trigger an innate immune response (Figure 2). However, dsRNA can be readily detected in JUNV-infected cells and co-localizes with PRRs (RIG-I and PKR) [54], which is consistent with the robust IFN/PKR responses observed in JUNV infected cells. Therefore, the role of NP ExoN in the context of virus infection remains to be examined in future studies. It is possible that the NP ExoN activity or its regulation is different among arenaviruses. It is also possible that the highly conserved NP ExoN may have an additional function(s) that is critical for arenaviruses. In addition to the ExoN activity, NPs of pathogenic arenavirus have been demonstrated to block IRF3 and NF-κB activation [72,73]. However, the IRF3 pathway does not appear to be completely blocked during NW arenavirus infection [60]. One possibility is that the IFN antagonist activities of the NPs of pathogenic NW arenaviruses (JUNV and MACV) are weaker than that of LASV NP during virus infection, explaining why the RIG-I/IRF3 signaling pathway is active in JUNV and MACV but not in LASV infection.

The NPs of non-pathogenic TCRV and MOPV degrade dsRNA in biochemistry assays. Expressed TCRV NP inhibits IFN reporter gene expression similarly to LASV NP [69]. Hence it seems that non-pathogenic and pathogenic arenaviruses sometimes have similar anti-IFN activities. It is worth noting that these data are based on assays with purified or plasmid-expressed NP. Nevertheless, TCRV or MOPV infection induces an IFN response [36,37,38,61]. During infection, the kinetics and the level of NP expression, as well as the distribution of NP are different from that in plasmid-expressed cells. Additionally, NP also engages in viral RNA replication, RNP formation, and virion formation. Therefore, the interaction of NP with the IFN pathway may be different in infected cells compared to that in plasmid-expressing cells, which may reconcile the difference sometimes observed in expression and infection studies.

Differences in interplay with PKR are also observed among arenavirus NPs. Unlike Candid#1 JUNV NP, neither LASV nor LCMV NP bind to PKR in co-expressed cells [66]. Co-localization of PKR with NW arenavirus NP can also be visualized in Candid#1 or TCRV infected cells [54,61]. Though more research is needed, the NPs of arenaviruses interact with the RIG-I and PKR pathways differentially, which may contribute to the differences in innate immune responses observed in clinical cases.

Recent research has also revealed that arenaviral NP inhibits the PACT-enhanced RIG-I-mediated IFN response [74]. PACT is a 313-amino-acid cellular protein that binds directly to both dsRNA and RIG-I [75] (Figure 2). EBOV VP35, IAV NS1, HSV-1 Us11, and MERS-CoV 4a and N, have been demonstrated to block PACT enhancement of the RIG-I-mediated IFN response by disrupting PACT and RIG-I interaction [76,77,78,79]. NPs of various arenaviruses (LASV, JUNV, MACV, TCRV, and PICV) inhibit PACT-enhanced, RIG-I-mediated IFN reporter gene expression [74]. This inhibition is not through blocking of PACT-RIG-I binding [74], but rather depends on the NP exonuclease activity, as both LASV and PICV NP mutants with defective exonuclease activity are unable to inhibit PACT-potentiated IFN-β production [74]. Thus, arenavirus NP is speculated to degrade dsRNA and thereby prevent PACT from binding to dsRNA. PACT is known to activate PKR [80], though the role of arenaviral NPs on PACT-mediated PKR activation remains unknown.

A recent large-scale protein interaction screen revealed that both OW and NW arenavirus NPs interact with the host DEAD-box ATP-dependent RNA helicase, DDX3 [81]. DDX3 exhibits a proviral role for arenaviruses, as replication of LCMV, LASV, and JUNV was substantially impaired in DDX3 knockout cells [81]. Two different mechanisms have been proposed for the proviral effect of DDX3. Early in infection, DDX3 assists in viral RNA replication, as supported by the reduced arenavirus minigenome replication in DDX3 knockout cells [81]. Mutation in the DDX3 ATPase and helicase domains also leads to reduced minigenome replication, indicating that these domains are essential for the proviral function of DDX3 [81]. Late in infection, DDX3 may act to suppress IFN production and facilitate LCMV infection [81]. By contrast, DDX3 does not affect IFN-β transcription in JUNV infection [81].

In addition to NP, arenavirus Z protein has also been implicated as a suppressor of the host innate immune response. Z has been shown to directly interact with RIG-I and prevent its association with MAVS, thus blocking IFN signaling [82,83,84] (Figure 2). Originally, differential roles for Z protein in OW and NW arenaviruses were assumed, as Z proteins of NW arenaviruses seemed uniquely capable of inhibiting RIG-I-mediated signaling [83]. However, more recent research has revealed that Z proteins of pathogenic arenaviruses, but not from nonpathogenic arenaviruses, are able to inhibit RIG-I and MDA-5 signaling [82]. The inhibition is mediated by interaction of the N-terminal domain on Z protein with the CARD domain on RLRs that disrupts RLR and MAVS interactions. The IFN antagonist activity of Z protein is found more prominently in macrophages, raising the possibility that Z proteins might inhibit the IFN response mainly in macrophages, while NPs act mainly in DCs. Further work will be needed to study the roles of Z protein in modulating the host immune response.

## 5. Arenavirus Subversion of Other Host Antiviral Defenses

The autophagy pathway is an important component of host antiviral defense, functioning to degrade pathogens and trigger both innate and adaptive immunity. While many viruses have evolved to suppress the autophagy pathway, others hijack it to assist in viral replication [85]. The role of autophagy during arenavirus infection is largely unknown, but recent research has provided evidence of a proviral role for autophagy. Two groups observed that both virulent and attenuated strains of JUNV induce autophagy early in infection [86,87]. Induction of autophagy prior to infection results in increased NP expression [87]. The OW MOPV also transiently induces autophagy during the first two days of infection, though LASV does not [88]. Nevertheless, depleting the essential autophagic vesicle protein ATG5 impairs MOPV, LASV, and JUNV replication [86,87,88], indicating that both OW and NW arenaviruses may utilize components of the autophagy pathway to aid in replication. While ATG5 is the key component in autophagy, there is increasing evidence that ATG5 also plays roles in non-autophagy processes, including negative regulation of RIG-I or MDA5 [89,90,91]. Thus, future studies are required to define if ATG5 is directly involved in arenavirus replication.

The mechanism behind autophagy induction in arenavirus infection is unclear. One group determined that, unlike replication-competent JUNV, UV-inactivated JUNV did not induce autophagy up to 9 hours post-exposure [86]. However, another group observed autophagy induction 24 hours after exposure to UV-inactivated JUNV [87], though it is unclear whether this was virus-driven. Autophagy likely plays a role late in arenavirus infection, as depleting ATG5 reduced extracellular, but not intracellular, LASV and MOPV RNA levels [88]. Several viral proteins have been demonstrated to interact directly with key components of the autophagy pathway. MOPV Z, and to a lesser extent LASV Z, interact with the sequestosome 1-like receptors NDP52 and TAX1BP1 [88]. JUNV NP has also been visualized co-localizing with LC3 [87]. However, the biological significance of viral protein interaction with components of autophagy remains unknown.

Another means by which pathogenic arenaviruses may subvert the host immune response may be through preventing or avoiding the activation of natural killer (NK) cells. NK cell activation is dependent on a mixture of activating and inhibiting signals presented by APCs [92]. It has been shown that LASV-activated macrophages are able to activate NK cells, but the activation is incomplete and ultimately does not suppress viral replication [93]. NP-mediated immunosuppression likely plays a role in preventing NK cell activation, as mutants lacking NP exonuclease function are able to fully activate NK cells in coculture with APCs [94]. Furthermore, it has been hypothesized that LASV may utilize NK inhibitory receptors to escape detection. LASV-activated NK cells are able to lyse K562 cells which lack HLA class-1 expression, but they cannot lyse LASV-infected DCs that are constitutively expressing HLA class-1 [93]. A recent study revealed that certain LASV epitopes bound to HLA-C1 molecules activate the KIR2DL2 inhibitory NK cell receptor, thus preventing NK cell activation [95]. Patients who succumb to LF are more likely to carry the KIR2DL2 gene than survivors [95]. The specific interactions between LASV bound to HLA molecules and the variety of inhibitory NK cell receptors may explain some of the clinical variability seen in LF cases.

## 6. Adaptive Immune Responses Following Successful Arenavirus Vaccination in Animal Models

For arenaviruses, the attenuated Candid#1 JUNV is the only vaccine that is approved for use in humans in Argentina. The vaccine elicits strong and protective neutralizing antibody responses in those vaccinated and has been successful in controlling AHF within endemic regions [96]. A chimeric MACV expressing the GPC from Candid#1 JUNV is highly attenuated in a mouse model and protects animals from lethal MACV challenge [97]. This protection correlates with high titers of MACV-specific neutralizing antibody before challenge.

Several LASV vaccine candidates currently in the preclinical stage are very promising based on their safety, immunogenicity, and efficacy in animal models. ML29 is a LASV/MOPV reassortant virus that contains the S segment of LASV and the L segment of MOPV. This vaccine platform expresses the main LASV antigens (GPC and NP). ML29 vaccination efficaciously protects NHPs and guinea pigs from LASV challenge through cell-mediated immunity; meanwhile the neutralizing antibody is below the detection level [98,99]. A recombinant vesicular stomatitis virus vector expressing the LASV GPC (VSV∆G/LVGPC) efficaciously protects NHP from lethal LASV challenge [100]. After VSV∆G/LVGPC immunization, a low level of neutralizing antibody is detected in vaccinated animals, meanwhile the T-cell response is measurable in one of four animals. Nevertheless, all vaccinated NHPs survived challenge and developed potent humoral and cellular immune responses. Finally, vaccination of NHPs with a vaccinia virus vector expressing LASV G1 and G2 confers protection from lethal LASV challenge [101]. The antibody response is low in survivors. Protection is very likely through cell-mediated immunity as vaccinia virus is known to induce the cellular immune response predominantly.

## 7. Impacts of the Adaptive Cellular Immune Response on Post-Infection Sequelae

Survivors of pathogenic arenavirus infection often develop post-infection sequelae in the months following the acute stage of illness. Neurological symptoms are common during NW arenavirus infection. This correlates with robust viral replication in neurological tissues as evidenced by high viral titers in the brains of infected animals [52,102]. MACV antigen can also be preferentially detected within neurons [52]. Interestingly, treatment of AHF and BHF with convalescent serum increases the likelihood of developing a long-term neurologic syndrome [43,103]. Whether neurological damage is directly caused by the virus infection or through an immune-mediated mechanism is still unknown.

In LASV infection, accumulating evidence has raised the possibility that the neurological sequelae are likely caused by virus-induced immunological injury. A recent study using NHPs as a model for LASV infection demonstrated that NHP survivors developed pathological findings consistent with autoimmune-associated vasculitis [104]. Two out of three NHP survivors also developed sensorineural hearing loss similar to that observed in human cases. Histopathological examination of the inner ear revealed inflammation of vessels and perivascular tissue at 45 days post-infection, long after the acute infection had subsided. Furthermore, serological analysis revealed that survivors developed elevated C-reactive protein and antineutrophil cytoplasmic antibodies, which are indicators of autoimmune disease [104]. These findings in NHPs indicate that hearing loss acquired after LF may be due to chronic inflammation.

Studies using a rodent model of LASV-induced hearing loss have provided further evidence that hearing loss may be caused by a cell-mediated immune response rather than through direct viral damage [105]. STAT1 knockout mice infected by clinical LASV isolates develop sensorineural hearing loss, though IFNαβ/γ receptor knockout mice do not. Interestingly, while LASV antigen can be detected in the inner ears of both types of mice, tissue damage was only observed in the STAT1 knockout mice concomitant with profound CD3-positive lymphocytic infiltration [105]. It would be interesting to determine if depletion of T cells could prevent hearing loss in this model.

One study using guinea pigs as a model for LASV infection determined that anterior uveitis was common during both fatal and nonfatal LASV infections [106]. This ocular inflammation was largely T-cell-mediated. However, low levels of LASV RNA were detected in the eyes of all guinea pigs who succumbed to infection as well as 3 of the 7 survivors. While viral antigen was not detected in the eye during this study [106], immunohistochemical staining has revealed the persistent presence of LASV in the smooth muscle of arteries in both a guinea pig and NHP model of infection, likely contributing to the long-lasting vasculitis [104,107]. Thus, it is currently hypothesized that persistently low levels of LASV replication trigger a chronic activation of the adaptive cellular immune response that leads to long-lasting inflammation.

## 8. Conclusions

Arenaviruses represent a continuing emerging threat as humans increasingly come into contact with their rodent reservoirs. Data from both clinical and animal model studies demonstrate that pathogenic OW and NW arenaviruses elicit vastly different immune responses, which have implications in viral pathogenesis. LASV infection is characterized by weak or delayed IFN-I/ cytokine induction and T-cell responses, while pathogenic NW arenaviruses (JUNV and MACV) trigger a robust IFN-I and pro-inflammatory cytokine response. Though the mechanisms behind these differences remain poorly defined, several observations have been noted. JUNV and MACV infections activate a variety of PRRs likely through dsRNA accumulation, while LASV seems to evade PRR detection. For all arenaviruses tested so far, arenaviral NP and Z proteins are capable of interfering with PRR activation and blocking innate immune signaling in expression studies. Nevertheless, their activity during viral infection remains to be determined. Differences in the innate immune response likely account for the differences seen in the adaptive response to hemorrhagic fever-causing arenaviruses. Overall, LASV clearance is associated with an early and strong cellular immune response, while recent findings have implicated the cellular immune response as a key contributor to the chronic inflammation and sequelae seen in LF survivors. This has profound implications in LASV vaccine development, particularly for those LASV vaccine candidates that are based on a T-cell response. Protection and recovery from pathogenic NW arenavirus infection are mediated by the humoral response. However, pathogenic NW arenaviruses that invade the immune-privileged central nervous system may evade clearance, which potentially causes neurological sequelae. This knowledge may inform the development of a neutralizing antibody-based therapy to treat AHF and BHF patients. Appreciation of the differential immune response to highly pathogenic NW and OW arenaviruses should facilitate the rational design of targeted therapeutics and vaccines.
